# Does COVID-19-specific news affect stock market liquidity? Evidence from Japan

**DOI:** 10.1016/j.mex.2023.102360

**Published:** 2023-09-04

**Authors:** Wurong Yang, Naoki Watanabel, Hideaki Sakawa

**Affiliations:** Graduate School of Economics, Nagoya City University, Nagoya, Japan

**Keywords:** Investor sentiment, OLS regression model, COVID-19 pandemic indices, Regression Analysis

## Abstract

This article examines the effect of COVID-19-specific news on stock market liquidity in the Japanese Topix 500-listed firms. Our empirical analyses show that both COVID-19 confirmed cases and COVID-19-specific news induce a negative effect on stock market liquidity. These findings suggest that the effect of COVID-19-specific news on U.S. stock market liquidity [1] is robustly confirmed in Japanese firms. This study also presents recommendations derived from Narayan et al. [2], who constructed a COVID-19-specific news index using data from popular newspapers worldwide. In sum, this study presents the following:

• Stock market liquidity is negatively affected by confirmed cases of COVID-19 in Japan.
• The impact of COVID-19-specific news on stock market liquidity was analyzed using the OLS regression method.

## Graphical abstract

**Figure:**
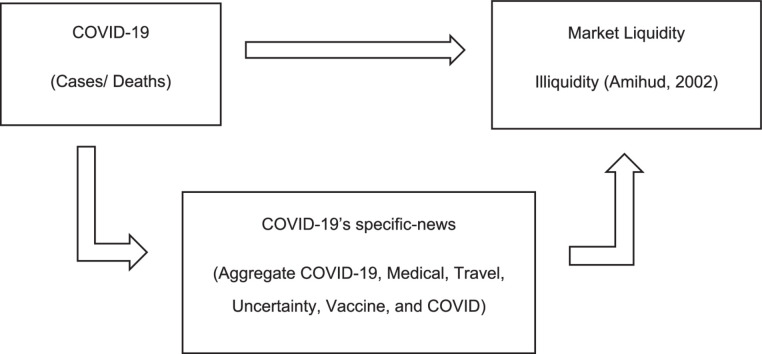

Specifications table

**Table:** 

Subject area:	Economics and Finance	
More specific subject area:	Financial Markets, COVID-19 Pandemic	
Name of your method:	Regression Analysis	
Name and reference of original method:	1. Dependent variable Amihud [3]. Illiquidity and stock returns: cross-section and time-series effects. Journal of Financial Markets 5(1): 31–56. https://doi.org/10.1016/S1386-4181(01)00024-6 2. Covid Index Narayan, P. K., Iyke, B. N., & Sharma, S. S. [2]. New Measures of the COVID-19 Pandemic: A New Time-Series Dataset. Asian Economics Letters 2(2). https://doi.org/10.46557/001c.23491	
Resource availability:	Our results can be reproduced by using econometric/statistical software such as Stata. Data can be retrieved from the following websites. The number of confirmed cases and deaths of Covid-19: https://covidtracker.bsg.ox.ac.uk/ Covid specific news indices: https://doi.org/10.46557/001c.23491	

**Method details**

## Introduction

This study aims to clarify how the COVID-19 pandemic affected stock market liquidity in Japanese financial markets. In financial markets, liquidity is an important factor during a pandemic, such as the COVID-19 pandemic [4]. In the U.S., liquidity equity markets deteriorated during the COVID-19 pandemic [1]. The negative investor sentiment induced by COVID-19-specific news affected stock market returns [2]. In fact, the confirmed cases of/deaths from COVID-19 reduced the stock returns [5]. With regard to stock market liquidity, previous studies found two channels through which it was affected. First, the confirmed cases or deaths negatively affected investor sentiment, which caused a decrease in stock market liquidity [1,6,7]. Second, the general sentiment related to COVID-19-specific news also negatively affected the stock market liquidity [1]. In this study, we aim to confirm the robustness of the effect of COVID-19-specific news [2] on stock market liquidity using the Japanese financial market setting.

Our research intends to fill the research gap of how COVID-19-related news affected stock market liquidity. During the first wave of COVID-19, stock market liquidity decreased with the increase of COVID-19 cases and negative sentiments in the U.S. equity markets [1]. In Japan, the negative news increased negative investor sentiment toward shipping firms [8] and restaurant firms [9]. Thus, the negative investor sentiment induced by COVID-19-specific news [2] also affected stock liquidity in Japan. However, research gaps regarding how COVID-19-related news affected stock market liquidity in Japan may remain.

We reveal how stock market liquidity was affected by the investor sentiment that arose following COVID-19-specific news. The WHO's declaration of the COVID-19 epidemic in China affected the Japanese stock market returns at an earlier stage of the pandemic [10,11]. Therefore, we conjecture that stock market liquidity was also affected by COVID-19-specific news. We used the OLS estimation method to gain the best linear unbiased estimator [12].

Our empirical results are summarized in the following three points. First, we determined that the increase in confirmed cases negatively affected the sentiment of market investors, resulting in decreased market liquidity. Second, COVID-19-related news also negatively affected the sentiment of market investors, and reduced market liquidity. Our study confirmed the robustness of previous studies that show that stock market liquidity was affected by COVID-19-specific news [1,7]. We also consider the possibility of non-linear estimators and estimate the non-linear regression, including that of the quadratic terms of COVID-19-specific news. Using non-linear regression, we also confirmed the robustness of our results.

## Data and methodology

### Data

Our sample consists of TOPIX 500-listed firms in Japan.^1^ Our sample period was January 23, 2020–April 28, 2021.^2^ The financial and stock return data of our sample firms were retrieved from their net profit margin (NPM) and the Astra Manager database. Data on the number of COVID-19 cases and deaths were retrieved from the Oxford Coronavirus Government Response Tracker (OXCGRT)^3^. We also obtained data from COVID-19-specific news indices [2], which included: the Aggregate COVID-19 index (A_Covid), Medical Index (Medical), Travel Index (Travel), Uncertainty Index (Uncertainty), Vaccine Index (Vaccine), and COVID Index (Covid).

**Fig. 1:**
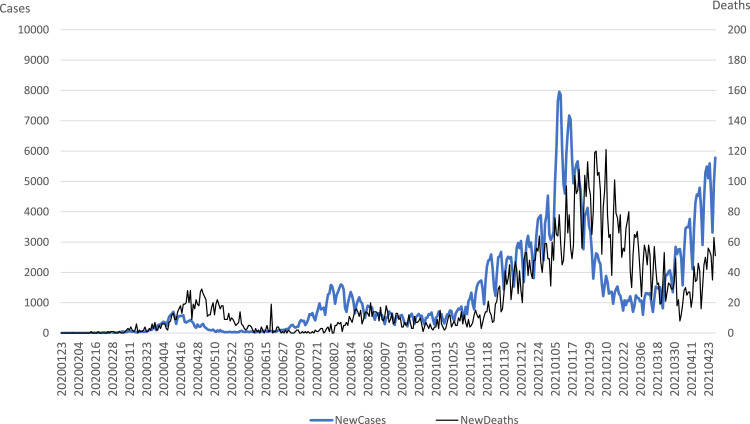
Number of daily cases and deaths in Japan.

Fig. 1 provides the time plot of Covid-19-related cases and deaths. During our sample period, new cases and deaths showed the highest value in October 2020 and increased again from March to April 2021.

### Methodology

We analyzed how stock market liquidity was affected by the investor sentiment that arose in response to COVID-19-specific news, following Baig et al. [1] and Haroon and Raviv [7]. We estimated the OLS regression Eq. (1) with robust standard errors clustering at firm levels [1]^4^. To control for the industry-specific effects, we included industry-fixed effects based on the Tokyo Stock Exchange's 33 industry sectors. To consider the robustness of the result, we also estimated non-linear regression as in Eq. (2).

(1) $$\text{ln}\;{\left(\text{liquidty}\right)}_{i,t}=\beta_{0}+\sum_{j}\beta_{j}\;\text{ln}{\left(\text{Pandemic}\;\text{Measures}\right)}_{i,t}+\sum_{j}\gamma_{j}{\left(\text{Controls}\right)}_{i,t}+\sum_{j}\delta_{j}\text{lndustr}y_{i}+\varepsilon_{i,t}$$

(2) $$\text{ln}\;{\left(\text{liquidty}\right)}_{i,t}=\beta_{0}+\sum_{j}\beta_{j}\;{\left(\text{Pandemic}\;\text{Measures}\right)}_{i,t}+\sum_{j}\theta_{j}{\left(\text{Pandemic}\;\text{Measures}\right)}_{i,t}^{2}+\sum_{j}\gamma_{j}{\left(\text{Controls}\right)}_{i,t}+\sum_{j}\delta_{j}\text{lndustr}y_{i}+\varepsilon_{i,t}$$

(3) $$ln\left({\text{Amihud}}^{\prime }s\;\text{Iiquidt}y_{i,t}\right)=\text{ln}\left(1+\frac{\left|Return_{i,t}\right|}{Volume_{i,t}}\right)$$

(4) $$ln\left(\text{Price}\;\text{Impac}t_{i,t}\right)=\ln\left(1+\frac{\left|Return_{i,t}\right|}{\frac{Volume_{i,t}}{\#\;of\;Outstanding\;shares_{i,t}}}\right)=\text{ln}\left(1+\frac{\left|Return_{i,t}\right|}{Turnover_{i,t}}\right)$$

To estimate Eq. (1), we investigated whether stock market liquidity was negatively affected by the COVID-19 pandemic-related indices in Japan. The dependent variable was the natural log of Amihud's [3] daily illiquidity measure (ln_illiq) as in Eq. (3) [1,7,15]. Illiquidity was intuitively interpreted as the daily price response related to one unit of trading volume and was also considered as a rough proxy of price impact [3]. To confirm the robustness, we also adopted the daily turnover price impact (ln_price impact) as an alternative illiquidity measure in Eq. (4) [16]. We adopted logs of the number of confirmed cases (ln_Case) and number of deaths (ln_Death) as measures of the pandemic, following previous studies [1,7,17]. In addition, we adopted the six indices proposed by Narayan et al. [2] as measures of the pandemic and used their natural logs as well [2]. We also controlled for the log of closing price of a stock (PRICE) and log of traded share volume (VOLUME). Volatility (VOLATILITY) was measured as the natural log of maximum price minus the natural log of minimum price [18]. Finally, we controlled for the daily return [1] using the daily return at the 1st section of the Tokyo Stock Exchange (TSE).

## Results

### Descriptive statistics

We present the descriptive statistics in Table 1. The mean value of illiquidity (ln_illiq) is 0.65. The means of ln_Case and ln_Death are 5.98 and 2.4, respectively. The mean of the aggregate index of COVID-19 (Ln_A_Covid) is 3.95. The means of COVID-19 specific news indices such as ln_Medical, ln_Travel, ln_Uncertainty, ln_Vaccine, and ln_Covid are 3.89, 3.23, 4.03, 3.37, and 3.84, respectively. With regard to the control variables, the mean closing price (PRICE) is approximately 4000 JPY, while the mean market capitalization (SIZE) is over 1100 billion JPY. The mean daily share volume (VOLUME) is approximately 2 million shares, and the mean volatility (VOLATILITY) is 0.026. Finally, the mean of the daily return at the 1st Section of the Tokyo Stock Exchange (TSE) is 0.048.

**Table 1:** Descriptive statistics.

Variable	Definition	Mean	Standard Deviation	Median	Q1	Q3
Ln_Illiq	The natural log of Amihud [3] illiquidity computed as the absolute returns divided by volume (scaled up by billion yen).	0.651	0.593	0.482	0.186	0.956
Ln_Price Impact	The definition is given in Eq. (4)	491.10	521.14	360.90	162.85	665.96
LnCases	The natural log of the number of people infected with coronavirus	5.978	2.018	6.438	4.585	7.415
LnDeaths	The natural log of the number of people who lost their lives due to coronavirus	2.433	1.396	2.565	1.386	3.664
LnA_Covid	Aggregate COVID-19 index (calculated in logarithm, [2])	3.953	0.217	3.941	3.887	4.032
LnMedical	Medical Index (calculated in logarithm, [2])	3.891	0.214	3.885	3.813	3.961
LnTravel	Travel Index (calculated in logarithm, [2])	3.228	0.516	3.252	3.025	3.511
LnUncertainty	Uncertainty Index (calculated in logarithm, [2])	4.032	0.301	4.046	3.933	4.159
LnVaccine	Vaccine Index (calculated in logarithm, [2])	3.366	0.586	3.255	3.074	3.866
LnCovid	COVID Index (calculated in logarithm, [2])	3.840	0.232	3.810	3.748	3.922
PRICE	The closing share price (Thousand, Yen)	4.004	6.280	2.471	1.422	4.335
SIZE	The market capitalization computed by multiplying closing price with shares outstanding (Billion, Yen)	1121.0	1988.2	478.0	246.0	1030.0
VOLUME	Share volume (Million)	2.010	6.474	0.733	0.316	1.754
VOLATILITY	The range-based volatility measure (the natural log of maximum price minus the natural log of minimum price [13]).	0.026	0.015	0.022	0.016	0.031
TSE	The daily returns series for the market index of Tokyo Stock Exchanges	0.048	1.344	0.089	−0.658	0.693

### Estimated results

We analyzed how the pandemic-related indices affected stock market liquidity. Table 2 depicts the results of the estimated OLS regression Eq. (1) in which illiquidity (Ln_illiq) is the dependent variable. First, we found that Ln_Case is significantly positively correlated to Ln_illiq. This result implies that an increase in confirmed cases of COVID-19 reduced liquidity in the Japanese stock market, consistent with Al-Awadhi et al. [5]. By contrast, we did not achieve significant results for Ln_Deaths. Second, we found that Ln_A_COVID is significantly positively correlated to Ln_illiq, suggesting that COVID-19 specific news negatively affected stock market liquidity. Each of the COVID-19 indices (i.e., Ln_Medical, Ln_Travel, Ln_Uncertainty, and Ln_Covid) also demonstrated a significant and positive correlation with stock market liquidity. Finally, Ln_Vaccine is significantly negatively correlated to Ln_illiq.

**Table 2:** Main empirical results.

Variable	(1)	(2)	(3)	(4)	(5)	(6)	(7)	(8)
	LnIlliq							
LnCase	0.0023*							
	(1.970)							
LnDeath		−0.0020						
		(−1.136)						
LnA_Covid			0.0844⁎⁎					
			(7.094)					
LnMedical				0.0965⁎⁎				
				(8.242)				
LnTravel					0.0177⁎⁎			
					(4.523)			
LnUncertainty						0.0251⁎⁎		
						(4.085)		
LnVaccine							−0.0131⁎⁎	
							(−3.123)	
LnCovid								0.1098⁎⁎
								(8.509)
PRICE	−0.0073*	−0.0073*	−0.0072*	−0.0072*	−0.0073*	−0.0073*	−0.0073*	−0.0072*
	(−1.996)	(−1.991)	(−1.984)	(−1.984)	(−1.988)	(−1.990)	(−1.987)	(−1.977)
SIZE	−0.0001⁎⁎	−0.0001⁎⁎	−0.0001⁎⁎	−0.0001⁎⁎	−0.0001⁎⁎	−0.0001⁎⁎	−0.0001⁎⁎	−0.0001⁎⁎
	(−3.702)	(−3.703)	(−3.709)	(−3.710)	(−3.705)	(−3.705)	(−3.704)	(−3.713)
VOLUME	−0.0139⁎⁎	−0.0139⁎⁎	−0.0139⁎⁎	−0.0139⁎⁎	−0.0139⁎⁎	−0.0139⁎⁎	−0.0139⁎⁎	−0.0139⁎⁎
	(−2.685)	(−2.692)	(−2.700)	(−2.700)	(−2.697)	(−2.693)	(−2.696)	(−2.709)
VOLATILITY	7.0974⁎⁎	7.0316⁎⁎	6.6636⁎⁎	6.6389⁎⁎	6.9398⁎⁎	6.9441⁎⁎	6.9864⁎⁎	6.4499⁎⁎
	(18.936)	(18.755)	(17.045)	(17.080)	(18.368)	(18.318)	(18.688)	(16.242)
TSE	−0.0029⁎⁎	−0.0025⁎⁎	−0.0039⁎⁎	−0.0044⁎⁎	−0.0023⁎⁎	−0.0027⁎⁎	−0.0020⁎⁎	−0.0040⁎⁎
	(−3.854)	(−3.346)	(−4.904)	(−5.514)	(−2.992)	(−3.487)	(−2.637)	(−5.106)
Constant	0.8565⁎⁎	0.8768⁎⁎	0.5469⁎⁎	0.5055⁎⁎	0.8169⁎⁎	0.7728⁎⁎	0.9172⁎⁎	0.4639⁎⁎
	(6.369)	(6.536)	(3.873)	(3.593)	(6.073)	(5.689)	(6.805)	(3.280)
Observations	153,610	153,610	153,610	153,610	153,610	153,610	153,610	153,610
R-squared	0.225	0.225	0.226	0.226	0.225	0.225	0.225	0.227
Industry FE	Yes	Yes	Yes	Yes	Yes	Yes	Yes	Yes

^a^ This table provides the results from the estimation of OLS regression model (1). All specifications include industry-fixed effects based on Industry classifications of the Tokyo Stock exchange. Robust standard errors are clustered at the firm level. T-statistics in parentheses.

⁎⁎ *p* < 0.01.

⁎ *p* < 0.05, + *p* < 0.1.

The robustness of results is confirmed by Table 3 which adopts liquidity (Ln_price impact) as the dependent variable. Using the estimate results of Table 3, we can gain almost similar results in Table 2. As for the vaccine-related news, we cannot gain the significant results.

**Table 3:** Robustness of results.

Variable	(1)	(2)	(3)	(4)	(5)	(6)	(7)	(8)
	Ln_Price impact							
LnCase	4.91⁎⁎							
	(4.152)							
LnDeath		2.85						
		(1.616)						
LnA_Covid			26.78*					
			(2.341)					
LnMedical				39.41⁎⁎				
				(3.565)				
LnTravel					5.79			
					(1.619)			
LnUncertainty						−7.45		
						(−1.142)		
LnVaccine							3.34	
							(0.858)	
LnCovid								47.15⁎⁎
								(3.968)
PRICE	−2.06	−2.04	−2.01	−2.00	−2.01	−2.03	−2.03	−1.98
	(−1.277)	(−1.265)	(−1.244)	(−1.241)	(−1.247)	(−1.257)	(−1.258)	(−1.230)
SIZE	0.02*	0.02*	0.02*	0.02*	0.02*	0.02*	0.02*	0.02*
	(2.310)	(2.316)	(2.318)	(2.316)	(2.322)	(2.320)	(2.318)	(2.317)
VOLUME	−8.22⁎⁎	−8.24⁎⁎	−8.25⁎⁎	−8.24⁎⁎	−8.25⁎⁎	−8.24⁎⁎	−8.24⁎⁎	−8.24⁎⁎
	(−2.605)	(−2.613)	(−2.624)	(−2.626)	(−2.623)	(−2.617)	(−2.616)	(−2.632)
VOLATILITY	6241.23⁎⁎	6189.41⁎⁎	6029.77⁎⁎	5983.97⁎⁎	6116.33⁎⁎	6187.38⁎⁎	6171.86⁎⁎	5893.98⁎⁎
	(19.177)	(19.018)	(17.239)	(17.311)	(18.624)	(18.542)	(19.015)	(16.531)
TSE	4.34⁎⁎	4.62⁎⁎	4.50⁎⁎	4.18⁎⁎	5.00⁎⁎	4.88⁎⁎	4.69⁎⁎	4.29⁎⁎
	(4.736)	(5.063)	(4.888)	(4.534)	(5.465)	(5.306)	(5.107)	(4.643)
Constant	112.77*	136.41⁎⁎	41.14	−5.30	126.31*	173.43⁎⁎	132.51⁎⁎	−30.91
	(2.291)	(2.801)	(0.653)	(−0.086)	(2.563)	(3.251)	(2.633)	(−0.490)
Observations	153,610	153,610	153,610	153,610	153,610	153,610	153,610	153,610
R-squared	0.073	0.073	0.073	0.073	0.072	0.072	0.072	0.073
Industry FE	Yes	Yes	Yes	Yes	Yes	Yes	Yes	Yes

^a^ This table provides the results from the estimation of OLS regression model (1). All specifications include industry-fixed effects based on Industry classifications of Tokyo Stock exchange. Robust standard errors are clustered at the firm level. T-statistics in parentheses.

⁎⁎ *p* < 0.01.

⁎ *p* < 0.05, + *p* < 0.1.

We further analyzed the non-linear regression, as in Eq. (2). Using the estimated results of Table 4, we confirmed results similar to those presented in Table 2. We also achieved significant and positive results for vaccine-related news, but negatively significant results related to the quadratic terms of vaccine-related news.

**Table 4:** Robustness of results (non-linear).

	(1)	(2)	(3)	(4)	(5)	(6)	(7)	(8)	(9)	(10)	(11)	(12)
	Ln_illiq						Ln_Price impact					
A_Covid	0.004⁎⁎						4.078⁎⁎					
	(7.927)						(7.242)					
A_Covid ^2	−0.000⁎⁎						−0.032⁎⁎					
	(−4.417)						(−6.312)					
Medical		0.006⁎⁎						5.315⁎⁎				
		(10.594)						(9.727)				
Medical^2		−0.000⁎⁎						−0.043⁎⁎				
		(−7.992)						(−9.422)				
Travel			0.002⁎⁎						1.677⁎⁎			
			(6.274)						(5.444)			
Travel^2			−0.000⁎⁎						−0.029⁎⁎			
			(−7.724)						(−8.880)			
Uncertainty				0.001						0.490		
				(1.350)						(1.028)		
Uncertainty^2				0.000*						−0.002		
				(2.060)						(−0.417)		
Vaccine					0.001⁎⁎						2.638⁎⁎	
					(4.253)						(8.233)	
Vaccine^2					−0.000⁎⁎						−0.036⁎⁎	
					(−10.276)						(−12.998)	
COVID						0.009⁎⁎						7.815⁎⁎
						(14.472)						(13.100)
COVID^2						−0.000⁎⁎						−0.067⁎⁎
						(−12.570)						(−13.267)
PRICE	−0.007*	−0.007*	−0.007*	−0.007*	−0.007*	−0.007*	−2.017	−2.019	−2.019	−2.010	−2.000	−2.004
	(−1.983)	(−1.985)	(−1.989)	(−1.981)	(−1.979)	(−1.979)	(−1.250)	(−1.252)	(−1.250)	(−1.246)	(−1.237)	(−1.241)
SIZE	−0.000⁎⁎	−0.000⁎⁎	−0.000⁎⁎	−0.000⁎⁎	−0.000⁎⁎	−0.000⁎⁎	0.015*	0.015*	0.015*	0.015*	0.015*	0.015*
	(−3.710)	(−3.710)	(−3.703)	(−3.709)	(−3.704)	(−3.711)	(2.318)	(2.315)	(2.329)	(2.321)	(2.329)	(2.324)
VOLUME	−0.014⁎⁎	−0.014⁎⁎	−0.014⁎⁎	−0.014⁎⁎	−0.014⁎⁎	−0.014⁎⁎	−8.244⁎⁎	−8.241⁎⁎	−8.228⁎⁎	−8.247⁎⁎	−8.223⁎⁎	−8.237⁎⁎
	(−2.702)	(−2.699)	(−2.693)	(−2.704)	(−2.703)	(−2.706)	(−2.621)	(−2.621)	(−2.614)	(−2.623)	(−2.623)	(−2.624)
VOLATILITY	6.616⁎⁎	6.642⁎⁎	7.056⁎⁎	6.664⁎⁎	6.970⁎⁎	6.555⁎⁎	6076.0⁎⁎	6032.7⁎⁎	6286.5⁎⁎	6087.7⁎⁎	6184.8⁎⁎	6075.3⁎⁎
	(16.519)	(16.912)	(18.162)	(17.042)	(18.627)	(16.069)	(16.820)	(17.196)	(18.450)	(17.392)	(18.941)	(16.460)
TSE	−0.004⁎⁎	−0.004⁎⁎	−0.003⁎⁎	−0.003⁎⁎	−0.003⁎⁎	−0.004⁎⁎	4.818⁎⁎	4.608⁎⁎	3.407⁎⁎	4.839⁎⁎	3.648⁎⁎	4.916⁎⁎
	(−4.825)	(−5.151)	(−4.423)	(−3.593)	(−3.925)	(−4.464)	(5.134)	(4.927)	(3.670)	(5.268)	(3.957)	(5.254)
Constant	0.718⁎⁎	0.679⁎⁎	0.837⁎⁎	0.820⁎⁎	0.867⁎⁎	0.604⁎⁎	24.685	−5.657	121.023*	123.654*	106.976*	−67.005
	(5.331)	(5.034)	(6.228)	(6.116)	(6.455)	(4.477)	(0.490)	(−0.113)	(2.471)	(2.468)	(2.178)	(−1.320)
Observations	153,610	153,610	153,610	153,610	153,610	153,610	153,610	153,610	153,610	153,610	153,610	153,610
R-squared	0.226	0.226	0.225	0.226	0.226	0.227	0.073	0.073	0.073	0.072	0.073	0.074
Industry FE	Yes	Yes	Yes	Yes	Yes	Yes	Yes	Yes	Yes	Yes	Yes	Yes

^a^ This table provides the results from the estimation of OLS regression model (1). All specifications include industry-fixed effects based on Industry classifications of the Tokyo Stock exchange. Robust standard errors are clustered at the firm level. T-statistics in parentheses.

⁎⁎ *p* < 0.01,.

⁎ *p* < 0.05, + *p* < 0.1.

## Conclusion

We investigated the relationship between stock market liquidity and the COVID-19 pandemic indices. First, we found that the number of confirmed cases decreased stock market liquidity. Second, COVID-19-related news lowered market liquidity by hampering the sentiment of market investors. Finally, vaccine-related news positively affected the market sentiment, and enhanced market liquidity; however, this result was not robustly confirmed.

Our study contributes to investors and policymakers under the threat of a pandemic, such as COVID-19. We found that public fear and an increase of COVID-19 cases deteriorated market liquidity. Thus, governmental restrictions would contribute to market liquidity.

These findings suggest two lessons to learn to ensure sufficient liquidity in the stock market. First, mitigation of the number of confirmed COVID-19 cases was important for the government to maintain a well-functioning market. Second, we found that the effect of COVID-related news affected market sentiment, which correlated to market liquidity. Overall, our results confirm the robustness of the effects of COVID-19-related investor sentiment on liquidity [1,7] in the Japanese stock market.

## CRediT authorship contribution statement

**Wurong Yang:** Software, Data curation, Writing – original draft. **Naoki Watanabel:** Methodology, Writing – review & editing. **Hideaki Sakawa:** Supervision, Conceptualization, Writing – review & editing.

## Declaration of Competing Interest

The authors declare that they have no known competing financial interests or personal relationships that could have appeared to influence the work reported in this paper.

## Data Availability

The authors do not have permission to share data.

## Footnotes

1 Baig et al. [1] adopted all the constituent stocks of the S&P 500. For comparability to U.S. markets, TOPIX 500 firms provide good reliability in Japanese markets [13]. Therefore, we focused on the TOPIX 500-listed firms.

2 Following Xiong et al. [14] and Sakawa and Watanabel [11], we started our analysis from January 23, 2020. Narayan et al. [2] provided data on COVID-19-specific news indices up to April 28, 2021.

3 OXCGRT: Oxford Coronavirus Government Response Tracker

4 We analyzed our data using Stata 18. To consider the correlation of error terms across firms, we estimated by using a “regression” command and “vce(cluster)” option. The “vce(cluster)” option specifies that standard errors allow for intragroup correlation within firms. In other words, it allows for observations that are independent across firms, but not necessarily within firms.
